# Supplementary figures and images for: Twin pregnancy and perinatal outcomes: Data from ‘Birth in Brazil Study’
Source: PLoS One. 2021 Jan 11;16(1):e0245152. doi: 10.1371/journal.pone.0245152 (PMC7799786; doi:10.1371/journal.pone.0245152)

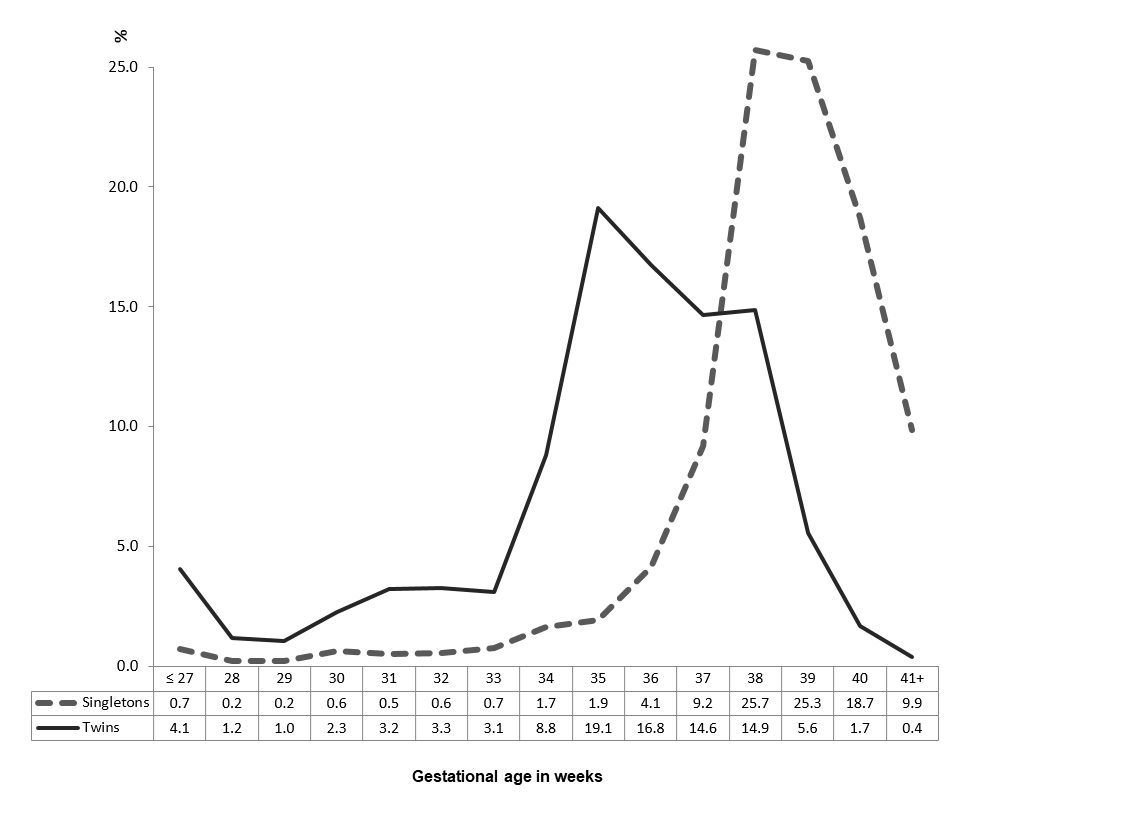

Supplement: S1 Fig — (TIF) [file pone.0245152.s001.tif]
