# Supplementary material for: Twin pregnancy and perinatal outcomes: Data from ‘Birth in Brazil Study’
Source: PLoS One. 2021 Jan 11;16(1):e0245152. doi: 10.1371/journal.pone.0245152 (PMC7799786; doi:10.1371/journal.pone.0245152)
Supplement: S1 Table — (DOCX) [file pone.0245152.s002.docx]

| **Supplemental table 1 - Onset of labour and mode of birth in twin and singleton newborns by gestational age groups** | | | | | | | | | | | | | | |
| --- | --- | --- | --- | --- | --- | --- | --- | --- | --- | --- | --- | --- | --- | --- |
| **^Onset of labour^** | **< 32 weeks** | | **32-33 weeks** | | **34-36 weeks** | | **37-38 weeks** | | **39-40 weeks** | | **>=41 weeks** | | **All** | |
|  | *Twins (n= 59)* | *Singletons (n=485 )* | *Twins (n= 39)* | *Singletons (n=266 )* | *Twins (n= 248)* | *Singletons (n= 1,813)* | *Twins (n= 164)* | *Singletons (n= 8,283)* | *Twins (n= 40)* | *Singletons (n=10,425)* | *Twins (n= 2)* | *Singletons (n=2,339)* | *Twins (n= 552)** | *Singletons (n=23,611)** |
|  | **n (%)** | **n (%)** | **n (%)** | **n (%)** | **n (%)** | **n (%)** | **n (%)** | **n (%)** | **n (%)** | **n (%)** | **n (%)** | **n (%)** | **n (%)** | **n (%)** |
| **Spontaneous** | 55.9 | 59.0 | 48.7 | 61.9 | 57.7 | 61.6 | 34.1 | 53.7 | 32.5 | 59.3 | 100.0 | 56.3 | 48.3 | 57.2 |
| Vaginal | 55.2 | 78.6 | 23.3 | 70.9 | 21.8 | 70.8 | 51.3 | 77.1 | 52.2 | 79.9 | 0.0 | 78.0 | 33.6 | 77.9 |
| Cesarean (IP) | 44.8 | 21.4 | 76.7 | 29.1 | 78.2 | 29.2 | 48.7 | 22.9 | 47.8 | 20.1 | 100.0 | 22.0 | 66.4 | 22.1 |
| **Provider-initiated** | 44.1 | 41.0 | 51.3 | 38.1 | 42.3 | 38.4 | 65.9 | 46.3 | 67.5 | 40.7 | 0.0 | 43.7 | 51.7 | 42.8 |
| Induction (VD) | 0.0 | 15.0 | 0.0 | 8.2 | 0.0 | 8.3 | 0.6 | 6.1 | 0.0 | 9.9 | - | 18.4 | 0.2 | 9.3 |
| Induction (CS) | 2.1 | 0.2 | 0.0 | 0.0 | 0.0 | 0.1 | 0.0 | 0.0 | 0.0 | 0.0 | - | 0.2 | 0.2 | 0.0 |
| Elective (CS) | 97.9 | 83.5 | 100.0 | 91.1 | 100.0 | 91.6 | 99.4 | 93.9 | 100.0 | 90.1 | - | 81.5 | 99.6 | 90.6 |
| * Information on the onset of labour was not available for two of the 554 twins and for 135 of the 23,746 singleton newborns (see table 1).  IP: intrapartum; VD: vaginal delivery; CS: Caesarean section | | | | | | | | | | | | | | |
